# Tumor-Associated Neutrophils and Desmoplastic Reaction in the Breast Cancer Tumor Microenvironment: A Comprehensive Review

**DOI:** 10.3390/cancers18030404

**Published:** 2026-01-27

**Authors:** Stavroula Papadopoulou, Vasiliki Michou, Arsenios Tsiotsias, Maria Tzitiridou-Chatzopoulou, Panagiotis Eskitzis

**Affiliations:** Department of Midwifery, School of Healthcare Sciences, University of Western Macedonia, Keptse, 50200 Ptolemaida, Greece; staupapadop@yahoo.gr (S.P.); atsiotsias@uowm.gr (A.T.); mtzitiridou@uowm.gr (M.T.-C.); peskitzis@uowm.gr (P.E.)

**Keywords:** breast cancer, tumor microenvironment (TME), tumor associated neutrophils (TANs), desmoplastic reaction (DR), extracellular matrix (ECM)

## Abstract

The evolving tumor microenvironment (TME) plays a critical role in breast cancer tumorigenesis, growth, and metastatic potential. This study focuses on two key components of the TME: tumor-associated neutrophils (TANs) and the desmoplastic reaction (DR). We will analyze their multifaceted functions, emphasizing the significant mutual relationships among them, which dramatically affect disease outcomes and the effectiveness of treatments. TANs can either suppress or promote tumors, demonstrating notable functional flexibility in response to signals from their immediate environment. Concurrently, the proliferation of myofibroblasts and the extensive deposition of extracellular matrix (ECM), which characterize the DR, substantially alter the tumor’s physical properties, increasing its stiffness. This increased stiffness significantly obstructs immune system cells from accessing the tumor, ultimately limiting the effectiveness of therapies and contributing to a more clinically aggressive tumor behavior. A comprehensive understanding of the interactions among TANs, the desmoplastic stroma, and other elements of the TME is critical for developing new predictive biomarkers and establishing more effective targeted therapies.

## Simple Summary

Breast cancer progression is strongly influenced by the tumor microenvironment, particularly by tumor-associated neutrophils (TANs) and the desmoplastic reaction (DR), a fibrotic stromal response. Increasing evidence shows that TANs and DR engage in a bidirectional, self-reinforcing interaction that promotes tumor growth, immune evasion, metastasis, and resistance to therapy. Pro-tumorigenic TANs enhance angiogenesis, extracellular matrix remodeling, and immunosuppression, while desmoplastic stroma increases tissue stiffness and limits immune cell infiltration and drug delivery. Significantly, immature desmoplastic patterns are associated with clinically aggressive breast cancer subtypes with poor prognosis and high TAN infiltration. This review highlights how TAN plasticity and stromal remodeling jointly shape disease progression and discusses emerging therapeutic strategies that target both immune and stromal components of the tumor microenvironment to improve outcomes in breast cancer.

## 1. Introduction

Breast cancer continues to represent a major global health burden and ranks among the most prevalent chronic diseases worldwide [1,2]. According to Global Cancer Statistics, BC has overtaken lung cancer to become the most frequently diagnosed cancer globally, with around 2.3 million new cases and approximately 670,000 to 685,000 deaths each year. This accounts for nearly 25% of all cancer diagnoses and 16–17% of cancer-related deaths among women [3]. Consequently, BC is the most common cancer among women in the majority of countries worldwide [4,5]. To be more precise, in 2022, Europe saw 557,873 new cases of female breast cancer, making it the most commonly diagnosed cancer among women in the region. This trend is reflected globally, with over 306,000 new cases reported in North America, primarily in the United States and Canada. Asia recorded approximately 985,000 cases, driven mainly by China and India, while nearly 200,000 new cases were reported in Africa. In Africa, the incidence of breast cancer is rising, along with ongoing regional disparities in detection and treatment outcomes [1]. Despite improvements in screening and treatment that have enhanced overall survival rates, 20–30% of patients still face unfavorable outcomes due to cancer subtypes with poor outcomes in the current therapeutic landscape and a high risk of relapse. Additionally, significant disparities in breast cancer incidence and mortality remain closely tied to levels of socioeconomic development. In countries with a high Human Development Index, approximately one in 12 women will be diagnosed with breast cancer during their lifetime, and one in 71 will die from the disease [6,7].

Breast cancer originates from the mammary epithelium, specifically the lobules and ducts embedded within fibrous and adipose tissue, and its development is strongly influenced by hormonal changes occurring during puberty, particularly in females [8]. While breast cancer can also occur in men, about 99% of cases are diagnosed in women, with only 0.5% to 1% occurring in males. Breast cancer is extremely heterogeneous and develops through complex interactions between genetic predisposition and environmental and lifestyle factors [4,5].

A recent prospective cohort study of adults under 50 years old from the UK Biobank has revealed that the risk of early-onset breast cancer is significantly increased due to higher polygenic susceptibility. Specifically, individuals in the highest risk tertile have an approximate hazard ratio (HR) of 3.06 compared to those in the lowest tertile (95% CI: 2.20–4.26). Furthermore, an unfavorable lifestyle further elevates this risk, particularly among those with the highest genetic susceptibility, where the hazard ratio is approximately 1.69. In contrast, the HR in the lowest genetic risk group is only 0.81 [9]. Polygenic risk refers to the susceptibility to disease that arises from the combined effects of many common genetic variants. Each variant may contribute only to a slight increase in risk, but together, they can significantly influence the development of diseases [10].

Individuals with more breast cancer risk alleles are more likely to develop the disease [11,12,13,14,15]. This mechanism differs fundamentally from rare hereditary cancer syndromes caused by single-gene defects, such as BRCA1/BRCA2-associated hereditary breast cancer [16], Li-Fraumeni syndrome (characterized by TP53 mutations) [17,18], or Nijmegen breakage syndrome [19], which have a high individual risk but low prevalence in the population. In breast cancer, polygenic risk is typically measured using polygenic risk scores (PRS). These scores integrate the weighted contributions of multiple single-nucleotide polymorphisms (SNPs) identified through extensive genome-wide association studies (GWAS) [15]. Unlike rare, high-penetrance mutations (such as BRCA1/2), polygenic risk reflects genetic diversity within the population and accounts for a significant portion of breast cancer heritability, currently estimated to exceed 30%. Large prospective studies have shown that PRS can effectively stratify women based on their lifetime risk of developing breast cancer [11,12,13,14,15]. Those in the highest percentiles of PRS are at several times greater risk compared to individuals with average or low genetic risk [14,15]. Importantly, PRS can be tailored to specific disease subtypes, such as estrogen receptor–positive or –negative breast cancer, which enhances risk prediction [20]. When integrated into clinical risk models, polygenic risk facilitates personalized risk assessment and supports targeted screening, prevention, and early detection strategies. This highlights the increasing importance of polygenic risk in predicting breast cancer risk and in public health planning [20].

Based on the above, a comprehensive understanding of its complex biology is imperative [21,22,23]. Research has increasingly concentrated on the role of the tumor microenvironment (TME) in breast cancer. The TME in breast cancer consists of cancer cells intertwined with immune, stromal, and vascular components, all within a dynamic extracellular matrix (ECM). This environment significantly influences tumor behavior, including local invasion, metastatic spread, and the response or resistance to therapy [21,22,23,24,25,26,27,28,29]. The cellular components of the TME include myeloid and lymphoid immune cells, fibroblasts, adipocytes, endothelial cells, and pericytes. Non-cellular components include abnormal blood vessels, ECM architecture, and soluble mediators. Together, these elements create a diverse and dynamic ecosystem that affects disease progression, immune evasion, and resistance to standard therapies [21,22,23,24,25,26,27,28,29,30]. Furthermore, TME plays a crucial role in shaping the immune response and tumor behavior through various signaling mechanisms. These mechanisms include cytokines, chemokines, growth factors, metabolic interactions, matrix remodeling, and biomechanical signals such as stiffness and hypoxia. This complex interplay promotes immunosuppression, enhances epithelial–mesenchymal plasticity, and establishes niches that facilitate metastasis spread and maintenance [31,32]. Consequently, these factors significantly impact the effectiveness of both standard treatments and emerging immunotherapies. Therefore, recent evidence positions the TME as an active driver of cancer progression and a potential therapeutic target in breast cancer [21,22,23,24,25,26,27,28,29].

In this review, we introduce the concept of the “tumor-associated neutrophils (TAN)–desmoplastic reaction (DR) axis,” which describes the dynamic, bidirectional communication between TANs and the DR within the breast cancer TME. This axis illustrates how TANs can promote fibroblast activation, ECM remodeling, and stromal stiffening, while also highlighting how the desmoplastic stroma can regulate neutrophil recruitment, polarization, and functional plasticity. Rather than functioning as a linear signaling pathway, the TAN–DR axis represents a reciprocal, self-reinforcing interaction between the immune system and the stroma that influences tumor progression, immune suppression, and therapeutic responses. Understanding this axis provides a foundational framework for exploring how the inflammatory and fibrotic components of the TME work together to affect the aggressive clinical behavior of breast cancer.

This review examines the role of two essential and interconnected components of the breast cancer TME: TANs and the DR. Its novelty focuses on their individual effects as well as their important synergistic influence on the progression of breast cancer.

## 2. TANs in Breast Cancer and Their Clinical Significance

Polymorphonuclear neutrophils, which originate from hematopoietic stem cells in the bone marrow, are primarily recognized for their strong phagocytic and bactericidal abilities. They have a central role in coordinating acute inflammation [33]. As the most abundant circulating granulocytes, neutrophils readily migrate to sites of tissue injury, infection, and neoplastic growth, accounting for their frequent presence in tumor tissues [21,22,23,24,25,28,34]. Within the TME, these infiltrating neutrophils, termed TANs, acquire context-dependent functions that extend beyond host defense and actively influence cancer progression [21,22,23,24,25,26,28,34,35]. TANs facilitate the formation of new blood vessels (angiogenesis), enhance cancer cell movement (motility), and modify the immune landscape. They often act as an immunosuppressive mechanism, influencing the behavior of other immune cell populations and promoting tumor progression [36]. Although research on neutrophil biology in cancer is complex and ongoing, there is a growing consensus that TANs are dynamic and adaptable contributors to the TME formation, and significant drivers of disease progression [34]. As a result, they have attracted substantial research attention.

### 2.1. Clinical Associations of TANs in Breast Cancer

A high number of TANs in the breast TME is often linked to negative prognostic indicators and poorer clinical outcomes. This is particularly true for triple-negative breast cancer (TNBC), a subtype that is known for having limited approved endocrine or HER2-targeted treatment options rather than an inherently higher level of biological malignancy. TNBC is typically recognized for its clinically aggressive nature and behavior [22,23,28,35,37]. Numerous studies have established a connection between high TAN density and adverse clinicopathological features of the tumor, such as larger tumor size, higher histological tumor grade [which reflects the degree of tumor cell differentiation and morphological atypia under the microscope and defined by the Nottingham Combined Histologic Grade (Elston–Ellis Modification) [38] based on tubule formation, nuclear atypia, and mitotic activity, lymph node involvement, and advanced disease stage [22,23,25,28,34,37]. Furthermore, a higher density of TANs is frequently associated with lower disease-free and overall survival rates in breast cancer patients [22,23,28,34,35,37]. In breast cancer subtypes, such as TNBC, TANs are commonly present and play a significant role [39]. They contribute to the immunosuppressive and metastatic characteristics of these tumors [21,22,23,25,28,35,37]. Interestingly, a recently published article found that, in TNBC, TAN-linked genes, such as the protumorigenic *RASGRP4*, *TIMM10B*, and *GRAP*, versus the tumor-suppressive TNFRSF13C, associated with poorer survival and represent actionable biomarkers and therapeutic targets [40].

Additionally, the absence of hormone receptors, specifically estrogen receptor (ER−) and progesterone receptor (PR−), strongly predicts TAN infiltration. This correlation links TANs with breast cancer subtypes that tend to have poorer clinical outcomes [21,22,23]. Consistent with this, increased TAN density has been reported in the ΤΜΕ of TNBC compared with hormone receptor–positive (HR+) tumors [41,42], whereas HR+ breast cancers typically display lower neutrophil infiltration [41]. However, it is essential to emphasize that TNBC’s classification as a clinically “aggressive” subtype largely reflects its lack of approved endocrine or HER2-targeted therapies rather than an inherent greater malignant potential. Indeed, hormone receptor–positive and HER2-positive breast cancers can also exhibit aggressive biological behavior in the absence of targeted treatment, and their improved prognosis is primarily attributable to the availability of effective endocrine and anti-HER2 therapeutic interventions [43]. In contrast, TNBC remains unresponsive to these targeted approaches and relies predominantly on systemic chemotherapy in the neoadjuvant or adjuvant setting [44]. Although TNBC patients are more likely to achieve a pathological complete response following neoadjuvant chemotherapy than those with luminal subtypes, individuals with residual disease experience particularly poor outcomes [44,45].

### 2.2. Plasticity and Dual Role of TANs

TANs exhibit considerable functional plasticity, adjusting their behavior in response to local TME signals rather than changing autonomously. Influences such as hypoxia, inflammation, tissue damage, and stromal remodeling affect neutrophil activity, modifying their functions and appearance through a process known as cytokine-guided polarization. This context-dependent activation results in two broadly defined functional states: anti-tumorigenic N1-like and pro-tumorigenic N2-like phenotypes [21,22,23,24,25,26,29,34,35]. Research by Fridlender et al. [46] in animal models highlighted this functional dichotomy, demonstrating that neutrophils infiltrating tumor sites adopt distinct activation states in response to local microenvironmental factors, rather than representing fixed differentiation lineages. Notably, high levels of transforming growth factor-β (TGF-β), often released during tissue repair, fibrosis, and hypoxia-associated inflammation, favor polarization toward an N2-like, pro-tumorigenic state. Importantly, these phenotypic states are dynamic and reversible, reflecting adaptive responses to evolving microenvironmental conditions rather than permanent lineage commitment [46,47].

Neutrophils constitute approximately 50–70% of circulating leukocytes and function as a primary component of the innate immune response [48]. Upon recruitment of tumors, they represent a rapidly mobilized population of effector cells whose activity is dictated by the TME’s inflammatory and metabolic context. Under conditions of acute inflammation and immunostimulatory signaling, neutrophils may support anti-tumor immunity and cytotoxic responses (N1-like). In contrast, chronic inflammation, hypoxia, ECM remodeling, and pro-fibrotic signaling promote immunosuppressive, pro-angiogenic, and pro-metastatic neutrophil functions characteristic of N2-like TANs. Thus, the TME not only governs neutrophil recruitment but also actively shapes their functional state through cytokines, growth factors, and mechanochemical signals, enabling context-dependent engagement of either anti-tumor or pro-tumor pathways [46,47].

### 2.3. N1 TAN Phenotype (Anti-Tumorigenic)

N1 TANs represent an anti-tumorigenic activation state of neutrophils that exists in a dynamic balance with pro-tumor N2-like TANs and plays a critical role in shaping cancer outcomes [49,50]. Rather than constituting a fixed lineage, the N1 phenotype reflects neutrophil activation in pro-inflammatory, immunostimulatory tumor microenvironments. Functionally, N1 TANs are pro-inflammatory and cytotoxic. They produce reactive oxygen species (ROS) and cytokines, such as tumor necrosis factor-α (TNF-α) interleukin (IL) 1β (IL-1β) and 6 (IL-6), that activate cytotoxic T cells, natural killer (NK) cells, and other components of the immune system. Through these mechanisms, N1-like TANs contribute to direct tumor cell killing and facilitate the recruitment and amplification of antitumor immune responses within the TME [21,22,23,24,26,27].

**Figure 1:**
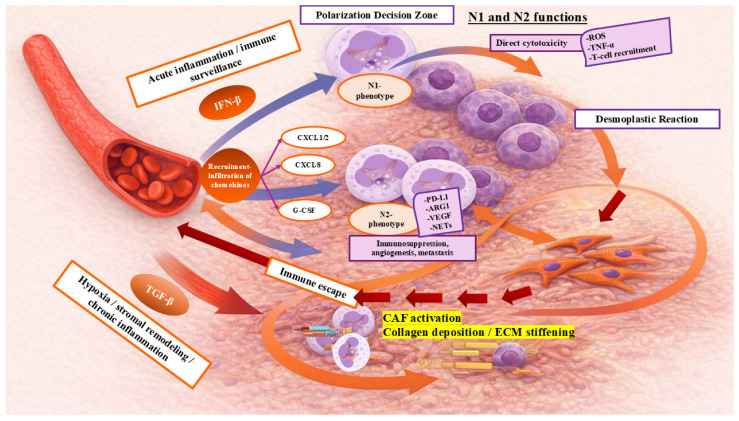
Cytokine-Driven Functional States of TANs in Breast Cancer. This schematic illustrates the recruitment of circulating neutrophils into breast tumors and their context-dependent functional activation within the tumor microenvironment (TME). Upon tumor infiltration, neutrophils are exposed to distinct local cytokine milieus that shape their behavior. Pro-inflammatory signals, including interferon-β (IFN-β), promote an N1-like activation state, characterized by enhanced cytotoxic activity, tumor cell killing, and support of anti-tumor immunity through recruitment and activation of T cells. In contrast, a TGF-β-rich, hypoxic, and tissue-remodeling microenvironment favors an N2-like functional state, associated with immunosuppression, pro-angiogenic signaling, ECM remodeling, and facilitation of metastatic progression. The figure emphasizes that TAN phenotypes reflect adaptive responses to local inflammatory and stromal cues, highlighting how the cytokine composition of the TME critically determines whether neutrophils support antitumor immunity or contribute to tumor progression.

In line with these functions, a higher presence of N1-like neutrophils, especially in the context of immunomodulatory or immune checkpoint therapies, has been linked to increased cytotoxic immune activity, decreased metastatic potential, and better survival outcomes [51,52]. These observations highlight the therapeutic potential of modifying the TME to promote N1-like neutrophil activation, instead of targeting neutrophils indiscriminately, especially in treatment-resistant breast cancer settings [52]. Mechanistically, N1-polarized TANs secrete cytotoxic molecules that directly kill tumor cells and enhance antigen presentation, activating adaptive immune responses and reinforcing anti-tumor immunity. This anti-tumor activation state is further supported by cytokines like interferon-β (IFN-β) and TNF-α, which emerge in inflammatory, immune-activating microenvironments and skew neutrophils toward an N1-like functional profile [21,22,23,24,25,26,34,35,53] (Figure 1). In breast cancer, multiple studies indicate that higher levels of N1-like neutrophil activity correlate with more favorable clinical outcomes, reflecting their capacity to restrain tumor growth and strengthen immune surveillance [54,55].

#### 2.3.1. The Association of Neutrophil Extracellular Traps (NETs) with N1-TAN Phenotype

N1-TANs exert potent antitumor effects through direct cytotoxicity, reactive oxygen species (ROS)–mediated interactions, and the formation of NETs, which together contribute to tumor cell killing and modulation of the tumor microenvironment’s immune landscape [56]. Beyond their direct effects on cancer cells, NET-producing N1 TANs participate in dynamic crosstalk with other immune populations, particularly macrophages and lymphocytes, through the release of cytokines and immunoregulatory mediators that shape local inflammatory and immune responses [57,58].

##### NETs in Breast Cancer

Neutrophils are the body’s rapid first responders to infections, environmental challenges, and tumor development [59]. Upon activation, they utilize three main effector mechanisms: phagocytosis, degranulation of cytotoxic enzymes, and the release of NETs through a process called NETosis. NETs are extracellular structures composed of decondensed chromatin that capture targets. They are enriched with proteins from the nucleus, granules, and cytosol, including neutrophil elastase (NE), myeloperoxidase (MPO) [60], histones, and other proteins such as S100A8/A9/A12, actin, and α-actinin [61,62]. By trapping invading pathogens within these DNA-protein lattices, NETs aid in their degradation and clearance by phagocytes. The proteolytic components of NETs include cathepsin G and other active enzymes that enhance antimicrobial and antitumor activities. Current research indicates that at least two primary pathways regulate NET formation, highlighting the mechanistic diversity of NETosis and its context-dependent regulation [63].

Stimuli like potent activator phorbol 12-myristate 13-acetate (PMA) and extracellular microbes activate the Suicidal (lytic) NETosis pathway. It involves the PKC–Raf–MEK–ERK signaling pathway and the activation of NADPH oxidase (NOX), leading to ROS generation in neutrophils [64,65,66,67,68]. ROS promotes the release of NE from granules and its nuclear translocation, resulting in chromatin decondensation. MPO then binds to chromatin and, together with NE, induces plasma membrane rupture and NET formation [60,69]. In contrast, the vital (non-lytic) NETosis pathway is a rapid process that does not involve cell death. In this process, nuclear chromatin is expelled along with granule proteins through degranulation. It can be triggered within minutes by activated platelets, microorganisms such as Staphylococcus aureus, and complement proteins [70,71,72]. This pathway is independent of NOX, and DNA-containing nuclear vesicles are extruded without disrupting the plasma membrane, thereby preserving neutrophil function [68,73].

Initially, these structures were thought to serve solely as a defense mechanism against microbes. However, they are now recognized as significant contributors to the progression and spread of breast cancer. In breast cancer, NET formation has been strongly associated with tumor progression and metastatic dissemination. NETs contribute to ECM remodeling by providing a scaffold enriched in proteases and matrix-modifying enzymes, thereby facilitating tumor cell migration and invasion. Although NETs may exert tumoricidal effects in specific experimental contexts, accumulating evidence indicates that, in the current therapeutic landscape, NET formation is predominantly linked to adverse clinical outcomes and pro-metastatic behavior [23]. Additionally, patients with metastatic breast cancer typically exhibit higher levels of circulating NETs compared to those with localized disease [74,75]. The pro-metastatic function of NETs is recognized at multiple stages of cancer spread [74]. They can specifically increase blood vessel permeability, allowing circulating tumor cells (CTCs) to escape from the bloodstream [60,63,76]. These web-like structures can also act as traps, physically entrapping CTCs and promoting the establishment of micro-metastases in distant organs such as the liver [76]. Furthermore, NETs can promote EMT in the cancer cells, thus making them more invasive [60,63]. For instance, in vitro, exposure to NETs increases N-cadherin and fibronectin levels while decreasing E-cadherin in breast cancer cells and causes morphological changes typical of EMT [74]. Since EMT is associated with cancer stemness, NETs also promote a CD44^high^/CD24^low^ phenotype in MCF7 cells in breast cancer, which is consistent with the acquisition of stem cell-like properties [77]. They also assist in establishing a pre-metastatic niche that prepares distant organs for tumor colonization [60,78].

An important feature of NETs is also their ability to “awaken” cancer cells that are dormant [60]. NETs help reactivate dormant cancer cells by remodeling the ECM. Proteases in NETs, such as NE and MMP9, cleave laminin, exposing a binding site that activates integrin α3β1. This activation triggers the FAK/ERK/MLCK/YAP signaling pathway, leading to the reawakening and proliferation of cancer cells [79,80], and thus leading to relapse of the disease [60]. Some authors characterize that NETs contribute to the “soil” that reactivates dormant disseminated cells, fostering a permissive microenvironment that supports the colonization and outgrowth of breast cancer “seeds” [81]. Still, in breast cancer, NET formation promotes lung metastasis and is associated with adverse prognosis [82].

Emerging therapeutic strategies aimed at disrupting NETs or the process of NETosis are being explored as adjuncts to reduce breast cancer spread [60,83]. One example is the dietary flavonoid kaempferol, which reduces ROS produced by NOX. This action helps to limit NETosis and lowers pro-metastatic signals [60,63,84]. Other approaches focus on targeting NET structure or on upstream neutrophil activation. For instance, the serine protease inhibitor elafin inhibits NE, destabilizing the NET scaffold and its proteolytic content [85]. Additionally, the CXCR1/2 antagonist reparixin reduces neutrophil recruitment and activation, thereby decreasing NET formation within the TME [86], while the enzyme DNase I degrades NETs DNA [87]. Together, these strategies represent distinct mechanisms to mitigate NET-driven metastatic progression [83].

### 2.4. N2 TAN Phenotype (Pro-Tumorigenic)

**Figure 2:**
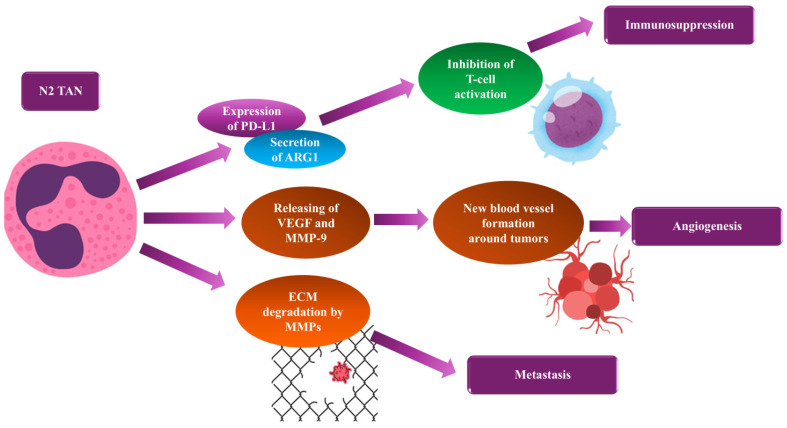
Pro-tumorigenic Mechanisms of N2 TANs. On the left, an N2 TAN directs three main processes, immunosuppression, angiogenesis, and metastasis, toward key targets illustrated on the right. For immune evasion, N2 TANs express PD-L1 and secrete ARG1, which inhibit T-cell activation and reduce their effector function. Regarding vascular remodeling, they release VEGF and MMP-9, which promote the formation and maturation of new blood vessels around the tumor. In terms of invasion and distant spread, N2 TAN-derived matrix metalloproteinases facilitate the degradation of the ECM, creating favorable pathways for tumor cell migration and metastatic spread. The arrows illustrate the direction of influence from the neutrophils to T cells, tumor vasculature, and the ECM. This highlights how the programming of N2 TANs coordinates immune evasion, vascular support, and matrix remodeling to promote disease progression.

TANs in an N2-like functional state mainly exhibit immunosuppressive and pro-tumor activities in response to specific microenvironmental cues, which promote tumor progression and metastatic spread [21,22,23,24,25,26,53] (Figure 2). Functionally, N2 TANs secrete a spectrum of mediators, including TGF-β (Figure 1), vascular endothelial growth factor (VEGF), and IL-10, among others. These mediators suppress antitumor immunity, increase tumor cell migration and invasion, and drive pathological angiogenesis [88]. Additionally, N2 TANs recruit and polarize regulatory T cells (Tregs) and myeloid-derived suppressor cells (MDSCs), further amplifying local immune evasion and creating a tolerogenic TME [88]. In treatment-naive breast cancer, an enrichment of N2-like neutrophil programs is associated with poor prognosis and resistance to both cytotoxic and immunotherapeutic interventions, especially in subtypes with a high inflammatory burden, such as TNBC [88,89]. In these settings, extensive tumor cell death, hypoxia, and ECM remodeling promote the release of inflammatory mediators and growth factors that favor N2-associated functional responses, thereby facilitating immune escape and metastatic spread. Research has also shown that knocking down CXCR2 in mouse breast cancer results in increased infiltration of TANs and a more pronounced pro-tumor N2 TAN spectrum, which promotes tumor growth and lung metastasis [90].

At the upstream level, TGF-β released from tumor cells, stromal fibroblasts, and damaged tissue acts as a dominant contextual signal driving N2-biased neutrophil responses within the TME. Additional inflammatory mediators, including granulocyte colony-stimulating factor (G-CSF) and IL-17, further reinforce this pro-tumor neutrophil state under conditions of sustained tissue injury, hypoxia, and chronic inflammation [21,22,23,24,25,26,28,34,35,89].

## 3. Mechanisms of TAN-Mediated Tumor Progression

The improvement of breast cancer progression by the N2 TANs is promoted through several mechanisms [22,23,26,35]:

- **Immunosuppression:** TANs suppress anti-tumor immunity by releasing mediators such as arginase-1 (Arg-1), ROS, and programmed death-ligand 1 (PD-L1) [22,23,24,34,91]. Arg-1 inhibits L-arginine, an amino acid essential for T-cell proliferation, thereby blocking T-cell activation [91]. Additionally, ROS directly impairs T-cell function, while PD-L1 expressed on TANs binds to PD-1 on T cells, leading to T-cell exhaustion [22,23,24]. Yet, N2 neutrophils contribute to a tolerogenic TME by releasing IL-10 and TGF-β. These factors inhibit the activity of cytotoxic T-cells and NK cells while promoting the recruitment and expansion of Tregs. This coordinated suppression of effective immune responses allows for unchecked tumor growth and is a key mechanism through which N2 neutrophils drive the progression of breast cancer [92].
- **Angiogenesis:** A malignant tumor definitely needs new blood vessels to grow, which TANs promote by producing a series of pro-angiogenic factors, such as VEGF, the peptide Bv8, and matrix metalloproteinase-9 (MMP-9) [21,22,23,24,25,28,34,53]. In addition, MMP-9 secretes growth factors that bind to the ECM and become accessible to endothelial cells [21,22,34].
- **Metastasis:** TANs play a crucial role in the invasion and spread of breast cancer. They enhance local tissue infiltration and facilitate the distant spread of neoplastic cells [1,2,3,4,5,8,10,11]. TANs secrete proteases that degrade the ECM, creating pathways for tumor cells to escape the primary tumor [21,22,23,24]. Additionally, they condition distant organs by releasing factors that prepare pre-metastatic niches to receive circulating tumor cells [22,23,24,34,53]. TANs also promote epithelial–mesenchymal transition (EMT), thereby increasing the motility and survival of tumor cells in circulation and at secondary sites [21,22,23,24,28,34,53]. Once tumor cells have disseminated, neutrophils that are skewed towards the N2 phenotype further enhance the formation of metastatic niches. They produce profibrotic cytokines that drive ECM deposition, remodel local tissue architecture, recruit immune subsets that support tumor growth, and sustain mechanisms of immune evasion, all of which facilitate the colonization and development of metastatic lesions [51,93].
- **Therapeutic Resistance:** Emerging evidence suggests that TANs, especially those with N2 phenotype, contribute to therapeutic resistance by protecting malignant cells from chemotherapy-induced death. They achieve this by sequestering or inactivating drugs and secreting mediators that activate pro-survival signaling pathways in tumor cells [22,23,24,28,34,35]. Moreover, to provide direct cytoprotection, N2 neutrophils enhance immune evasion in the TME, diminishing the effectiveness of immune-dependent therapies (such as checkpoint inhibitors) by continuously secreting immunosuppressive cytokines. Their remodeling of the TME, characterized by reduced permeability and altered stromal architecture, hinders drug penetration to malignant areas [23,24]. Together, these mechanisms promote tumor cell survival, undermine the effectiveness of cytotoxic and targeted therapies, and underscore the importance of targeting N2 TANs in strategies to overcome therapeutic resistance [94].

## 4. DR in Breast Cancer: Biological Basis and Clinical Impact

DR refers to the growth of fibrous or connective tissues at the sites where cancer invades the stroma. This reaction involves fibroblasts, lymphatic and vascular endothelial cells, immune cells, abnormally increased nerve tissue, and ECM. Together, these components create a complex TME that supports cancer development, invasion, metastasis, and resistance to anticancer therapies [95]. DR, or tumor-associated fibrosis, is commonly found in many solid tumors and in breast cancer [22,28,96,97,98], involving the transformation of fibroblasts into cancer-associated fibroblasts (CAFs) and myofibroblasts, as well as the massive deposition of ECM components, especially collagen [21,25,26,27,28,29,97,98,99,100,101]. In breast cancer, DR produces heterogeneous tissue mechanics and architecture, leading to uneven drug distribution and variable therapeutic response across patients, thereby sustaining elevated disease mortality [102]. In a pioneering study, the authors replicated the biomechanical microenvironment of breast cancer DR in vitro, which hinders drug delivery to tumors. Tumoroids were created using breast cancer cell lines (MCF7, SKBR3, MDA-MB-468) and fibroblasts in microfluidic devices with microvascular networks. The results showed that tumoroid human microvascular networks (MVNs) with more invasive cell types (SKBR3, MDA-MB-468) exhibited greater hyaluronic acid deposition and a higher proportion of fibroblasts transformed into CAFs than MCF7 tumoroids and control MVNs [102].

### 4.1. Classification of DR

The DR does not develop on its own; instead, it is actively triggered by tumor cells. Cancer cells produce ECM metalloproteinase inducer (EMMPRIN/CD147), which stimulates nearby stromal cells, especially cancer-associated fibroblasts, to generate MMPs and modify the ECM [103,104,105]. Through paracrine signaling, tumor cells coordinate the activation of fibroblasts, collagen remodeling, and stroma expansion. This process drives the DR, which aids tumor invasion and progression [106]. Recent research in breast cancer supports this mechanism. Fang Li et al. [105] demonstrated that CD147 (also known as EMMPRIN), which is derived from tumor cells, enhances processes such as migration, invasion, epithelial-mesenchymal transition, MMP-9 expression, and drug resistance. This occurs through activation of the MAPK/ERK pathway, illustrating a direct link between tumor-derived CD147 signaling and changes in the ECM that contribute to more aggressive tumor behavior. These findings identify CD147 as a key tumor-derived driver of stromal remodeling and a potential therapeutic target in breast cancer.

The DR shows great variety and can vary widely, from a dense, acellular fibrotic tissue to a cellular stroma with minimal collagen [22,97,99]. Histopathologic evaluation of the invasive front generally categorizes the DR into three morphological patterns: mature, intermediate (keloid-like), and immature (myxoid), based on the presence of myxoid stroma and hyalinized collagen in the invasive front of the tumor, the composition of the ECM and the architecture of the stroma, as observed in hematoxylin-eosin-stained sections. Each of these patterns has potential prognostic value [107,108]:

- **Immature DR (presence of myxoid stroma):** In this type of DR, the stroma is characterized by a large amount of mucinous material and loosely organized collagen fibers and may be found in clinically aggressive tumors [16,22]. In breast cancer, Wernicke et al. [109], found that stromal myxoid changes, along with elevated hyaluronan levels, are strongly associated with nodal positivity, higher tumor grade, and lymphatic emboli. This highlights a high-risk subset and reinforces the role of hyaluronan in breast cancer invasion and metastasis [109]. Extending the prognostic relevance of myxoid remodeling, Nearchou et al. [110] noted that, to their knowledge, no previous study had assessed the prognostic significance of the total myxoid stroma area at the extramural tumor front. Their analysis was the first to demonstrate a strong predictive value for colorectal cancer across both training and validation cohorts. In line with this, a prior study [111] found that the immature stromal group, characterized by the presence of myxoid change, had the poorest prognosis in colorectal cancer. These findings may also inform investigations into stromal myxoid remodeling in breast types like TNBC, potentially uncovering similar prognostic markers and mechanisms. Consistent with this direction, Yanai et al. [96] reported that myxoid change and FF are independent poor prognostic indicators in patients with TNBC who were receiving adjuvant chemotherapy (54.8%) or not (40.3%) and thus histopathologic evaluation of myxoid change and fibrotic focus (FF) in TNBC may serve as a practical, readily assessable prognostic tool, warranting validation in larger prospective cohorts. At this point it is crucial to note that neither myxoid change nor fibrotic focus, whether alone or in combination, was significantly associated with the presence of adjuvant chemotherapy. Lastly, they further emphasized the need to standardize diagnostic criteria for myxoid change and FF in TNBC and to elucidate the underlying molecular mechanisms.
- **Intermediate DR (presence of keloidal stroma):** This pattern is characterized by thick, hyalinized collagen bundles that look like keloid scars and is also generally considered as an immature and active stromal response to tumors [112]. In line with emerging evidence that stromal maturation modulates breast cancer behavior, Zhai et al. [113] reported a predominance of intermediate (keloid-like) DR (60.6%), with smaller proportions of immature myxoid (26.9%) and mature (12.5%) patterns. Importantly, in multivariable models, mature stroma was significantly associated with improved overall survival compared with immature stroma, whereas intermediate stroma conferred only a nonsignificant trend toward benefit. These findings position intermediate DR as prognostically superior to immature myxoid stroma but inferior to mature collagen-rich stroma, underscoring the clinical relevance of stromal grading and the potential utility of targeting stromal maturation in therapeutic strategies.
- **Mature DR:** In a mature DR, dense, well-organized collagen fibers are found, along with a more uniform cell distribution [107]. This type of DR may indicate a more contained or less clinically aggressive stromal response [107]. DR was once thought to be just a host response to a tumor [107]; however, now there is strong evidence that it can actually precede and promote cancer development, which is facilitated by creating a pro-tumorigenic environment [26,28,97,107]. Additionally, the mature group had the highest recurrence-free survival rates in patients with colorectal cancer [111] and breast cancer [113].

### 4.2. Mechanisms Driving DR: What Do We Now So Far?

A complex interaction between cancer cells and the surrounding stromal cells drives the development of DR [2,3,19]. This process involves the following crucial steps:

#### 4.2.1. Myofibroblast Activation

A notable variety of signaling molecules can be produced by the cancer cells, which include transforming growth factor beta-1 (TGF-β1) and platelet-derived growth factor (PDGF), which can actually be powerful signals to convert local fibroblasts into highly productive myofibroblasts [21,22,28,29,99]. On the other hand, these activated myofibroblasts are primarily responsible for the excessive deposition of ECM proteins, specifically collagens, fibronectin, and various proteoglycans [22,26,27,28,97,99,100,107]. In breast cancer, the activation of myofibroblasts, identified by the presence of α-SMA-positive CAFs, plays a crucial role in determining tumor behavior. This activation influences therapeutic responses, drug accessibility, and immune regulation, ultimately correlating with poor survival rates [114]. Activated fibroblasts are not only widespread and genetically stable across different malignancies, but they also contribute to creating a pro-tumor environment through processes such as ECM remodeling, paracrine signaling, and immune and vascular disruption [114].

Research by Hendrayani et al. [115] has shown that pre-treatment TNBC cells activate breast stromal fibroblasts in an IL-6–dependent manner via a STAT3→AUF1 signaling pathway. This leads to the downregulation of tumor suppressors p16, p21, and p53, while simultaneously increasing the levels of IL-6, SDF-1 (CXCL12), TGF-β1, and α-SMA. As a result, it establishes a self-reinforcing loop of fibroblast activation that supports tumor growth, suggesting that AUF1 and the upstream IL-6/STAT3 signaling could be viable targets for anti-metastatic therapies. Additionally, research by Aboussekhra et al. [116] indicates that a significant portion of activated breast stromal fibroblasts may acquire progenitor or stem-like characteristics. This suggests that the “activated CAF” state may be a stable cellular condition rather than a defined lineage, highlighting the potential for therapeutic approaches to normalize or reprogram these specific cell states.

#### 4.2.2. ECM Remodeling and Stiffening

The physical properties of a tumor are significantly influenced by ECM accumulation, leading to a noticeable increase in density and stiffness [22,23,29,98,100,101]. This increased stiffness is not merely a passive characteristic; it acts as an active mechanical signal that promotes the growth, resistance, and movement of cancer cells. This process often involves the activation of mechanosensors, such as integrins [22,25,29,97,98,100,101]. Additionally, tumor cells can secrete enzymes, such as MMPs, that allow them to navigate through this dense stroma. These enzymes can create openings within the stroma, enabling the tumor cells to infiltrate surrounding tissues and, importantly, to form new blood vessels [21,22,25,27,28,29,98,100,101].

In breast cancer, remodeling of the ECM, resulting from complex interactions between the tumor and the TME, alters matrix composition, architecture, and mechanics, creating a pro-invasive environment that influences tumor cell behavior [99]. Tumor formation is characterized by the interplay between ECM stiffening and proteolytic degradation, with increased rigidity in breast tumors primarily driven by collagen accumulation and abnormal cross-linking [117,118]. Studies on human breast tissues indicate that the progression from non-malignant epithelium to invasive ductal carcinoma is associated with significant collagen deposition and increased stiffness in the surrounding stroma [119]. Extending these observations mechanistically, Peng et al. [120], conducted further research that showed targeting the mechanics of the ECM can inhibit breast tumors. They found that metastatic matrices have wavier, finer fibers and increased stiffness, attributed to higher levels of type IV collagen, specifically COL4A2. This protein promotes tumor-cell migration and metastasis. In contrast, reducing COL4A2 levels in vivo leads to decreased ECM stiffness, reduced cell migration, and a lower metastatic burden. This suggests that targeting COL4A2-induced basement membrane stiffening could be a viable therapeutic approach [120].

### 4.3. Clinical Significance

Incorporating all of the above into a clinical theory, we can analyze the DR in the following ways:

- **Prognostic Indicator:** The specific characteristics of the stroma are significant prognostic indicators. For instance, immature forms of DR, such as myxoid or keloid-like stroma, are frequently associated with higher histological grade tumors and adverse clinical outcomes, particularly in breast cancer subtypes that lack effective targeted therapies, thereby contributing to a poorer prognosis in the current therapeutic landscape [22,28,96,107].
- **Therapeutic Implications:** The physical barrier created by DR presents a major challenge for therapy. The dense fibrous tissue associated with DR can hinder the effective delivery of chemotherapy and immunotherapies to the cancer cells within the tumor. The high interstitial fluid pressure further exacerbates this issue [22,25,26,27,28,29,96,100,121].

As a result, this barrier effect significantly reduces treatment efficacy, which can help explain the development of therapy resistance [22,26,96,100,121]. Additionally, CAFs not only act as a protective shield but can also release factors that enhance cancer cell resistance and survival or alter drug metabolism [22,26,100].

## 5. TANs and DR Within the Breast Cancer TME: Interactions

**Figure 3:**
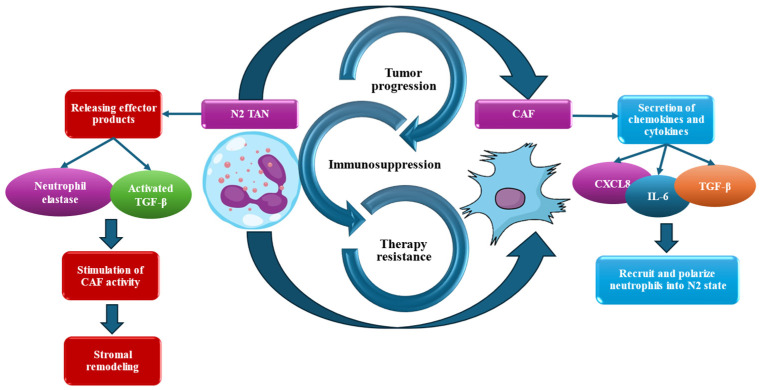
The TANs-desmoplasia vicious feedback loop feed. The figure illustrates the functional synergy within the TME, where TANs recruitment and N2 polarization, driven by CAFs signals and stiffness, lead to further ECM remodeling via MMPs and NETs. On the right side, CAFs secrete chemokines and cytokines, specifically CXCL8, IL-6, and TGF-β, which recruit and polarize neutrophils towards an immunosuppressive N2 state. On the left side, N2 TANs release effector products, such as neutrophil elastase and the activation of latent TGF-β, which further stimulate CAF activity and contribute to stromal remodeling. The large circular arrows highlight the feedback nature of these interactions, which lead to three key outcomes: tumor progression, immunosuppression, and therapy resistance. This feedback loop creates a physical and immunological barrier that facilitates immune escape and cancer progression.

The relationship between TANs and the DR should not be viewed as a simple one-way interaction; instead, it is a complex, self-sustaining, and destructive synergistic relationship. This bidirectional relationship is crucial for the development of cancer [122]. On one hand, the physical and chemical characteristics of the stroma dynamically affect the immune cells within it, including neutrophil polymorphonuclear cells. This influence impacts their location, type, and function [21,22,23,27,28,96,101]. On the other hand, these immune cells actively remodel their environment and play a significant role in the fibrotic process. As a result, a vicious feedback loop is established that ultimately benefits the tumor [100,122] (Figure 3).

### 5.1. ECM Stiffness and Mechanotransduction in Immune Cell Plasticity

Elevated matrix stiffness within the TME functions as a potent regulator of immune cell behavior by engaging mechanotransduction signaling pathways, thereby shaping TAN polarization toward a pro-tumorigenic state [123,124]. Immune cells actively sense and respond to changes in ECM rigidity via mechanosensitive receptors, enabling bidirectional crosstalk between matrix remodeling and immune regulation [125]. Increased matrix stiffness not only forms a physical barrier that restricts immune cell infiltration [126,127], but also acts as a biochemical instructor that induces immunosuppressive signaling programs [128]. Through integrin-mediated adhesion complexes, stiff matrices activate intracellular signaling cascades, such as PI3K/AKT, and promote cytoskeletal remodeling, facilitating the nuclear translocation of mechanosensitive transcriptional regulators, including YAP/TAZ [124,129]. Recent evidence suggests that neutrophil mecanosensing involves integrin-dependent cytoskeletal tension and FAK/SRC signaling, which cooperate with inflammatory cues to reinforce N2 polarization under conditions of increased matrix stiffness [123]. Meanwhile, mechanosensitive ion channels convert mechanical cues into calcium-dependent signaling responses that further modulate immune cell activation and differentiation [129]. Although most mechanistic insights have been derived from studies on macrophages, where matrix stiffness promotes immunosuppressive M2 polarization via integrin–FAK–cytoskeleton–nuclear coupling pathways, emerging evidence suggests that similar mechanotransduction mechanisms operate in TANs [123,130]. In stiff, desmoplastic matrices, neutrophils undergo persistent mechanical stress, which skews them towards an N2-like, immunosuppressive phenotype marked by reduced cytotoxicity and increased pro-tumor functions. It is now clear that matrix stiffness regulates TAN polarization through integrated physical and signaling mechanisms, reinforcing immune evasion and tumor progression [123,131].

### 5.2. DR as a Contextual Regulator of TAN Function

The DR exerts a dominant regulatory influence on TANs by shaping their recruitment, spatial distribution, and functional bias within the TME. Rather than directly initiating neutrophil-mediated immunosuppression, the desmoplastic stroma primarily conditions and stabilizes pre-existing tumor-promoting neutrophil programs, thereby reinforcing immune evasion and disease progression [21,22,23,100,122]. At the physical level, the dense ECM in desmoplastic tumors imposes mechanical constraints that limit immune cell infiltration and alter neutrophil trafficking [27]. Concurrently, CAFs, a key component of the DR, secrete chemokines such as CXCL1, CXCL2, and CXCL8, which actively attract neutrophils to the tumor site. This combination of mechanical exclusion and chemokine-driven recruitment leads to the accumulation of TANs in stromal-rich areas of the tumor, including breast tumor [21,22]. Once neutrophils are recruited, their functional states are further influenced by biochemical and biomechanical signals from stromal cells. Cytokines released by CAFs, such as IL-6, along with increased ECM stiffness, push TANs toward a pro-tumorigenic phenotype. This process amplifies the immunosuppressive signaling programs already present within the TME [21,101].

### 5.3. TANs as Active Architects of the DR

While the DR influences neutrophil behavior, TANs contribute to establishing and maintaining the fibrotic tumor architecture, creating a self-sustaining feedback loop [21,22,23,100,122]. TANs should therefore not be regarded solely as passive responders to stromal cues, but as active participants in stromal remodeling and fibroblast activation. Pro-tumorigenic TANs release a variety of mediators that promote fibroblast activation and ECM deposition, thereby strengthening the DR. Among these mediators, neutrophil elastase and MMP-9 play key roles in matrix reorganization, collagen turnover, and fibroblast stimulation, contributing to the expansion of fibrotic stroma [21,22,23,122]. In addition, TAN-derived cytokines such as TGF-β and CXCL8 directly promote the differentiation of resident fibroblasts into CAFs, while IL-6 sustains fibroblast activation through STAT3-dependent signaling pathways [47].

NETs derived from TANs have also been shown in preclinical models to stimulate CAF activation and increase ECM deposition, contributing to greater stromal stiffness and architectural complexity [132]. In the context of breast cancer, it is best to understand NET-associated activity as a factor in stromal remodeling, rather than viewing it as an isolated cause of tumor cell invasion [60]. This reciprocal relationship creates a reinforcing cycle in which stromal stiffening and fibroblast activation drive TAN polarization toward tumor-supportive states. As the matrix density increases, it enhances mechanical barriers to the infiltration of cytotoxic immune cells and maintains chemokine gradients that attract more neutrophils. This cycle perpetuates immune exclusion and fibrotic progression, emphasizing that the pathological impact of TANs and the DR arises from their dynamic, mutually reinforcing interactions within the breast cancer TME [46,47].

The transition to a mature, dense fibrous stroma further illustrates the complexity of this interplay. Here, tightly packed collagen fibers form a robust physical barrier that can prevent immune cell entry [23,101]. Nonetheless, the cells responsible for constructing this barrier continue to secrete chemokines that recruit neutrophils. At the same time, the environment’s extreme rigidity influences the movement and function of those TANs that successfully breach the barrier [21,23,101].

To summarize these findings, Table 1 presents a conceptual overview of the key bidirectional interactions between TANs and the DR. The table emphasizes how biochemical signals, such as TGF-β and IL-6, interact with biophysical properties, such as matrix stiffness [23,101]. A primary example is the feed-forward circuit in which TGF-β induces N2-like polarization; in turn, these N2 TANs secrete factors, such as neutrophil elastase, that further activate latent TGF-β [23,24,122]. Together, these factors create a sophisticated, context-dependent regulatory network that promotes an increasingly aggressive, pro-fibrotic environment.

### 5.4. Subtype-Specific Heterogeneity of TANs and DR in Breast Cancer

The composition and functional interactions between TANs and the DR vary significantly across breast cancer molecular subtypes, with direct implications for immune regulation and therapeutic response. Furthermore, evaluating the TME at the time of diagnosis may offer valuable insights into the host immune response to cancer as well as the tumor’s sensitivity to chemotherapeutic treatment [134]. Growing evidence indicates that the stromal and immune components play crucial roles in TNBC biology, treatment response, and prognosis. The transition from immune surveillance to immunoediting illustrates how the dynamic interactions between tumor cells and the immune system influence tumor elimination, equilibrium, and immune escape [135,136]. Although breast cancer is generally less immunogenic than “hot” tumors such as melanoma, TNBC represents the most immune-active subtype, exhibiting increased immune gene expression and higher densities of stromal and intratumoral tumor-infiltrating lymphocytes (TILs) [137,138]. TIL abundance, as observed in hematoxylin and eosin–stained sections, varies significantly across TNBC and is influenced by immune cell transcriptional programs, cytokine signaling (such as interferons), and various somatic, epigenetic, and germline alterations [139]. Importantly, TILs are an independent favorable prognostic factor in both chemotherapy-treated and treatment-naïve early TNBC [138,140].

A high infiltration of immune cells is typically observed in TNBC. This includes an increased density of TANs that predominantly display an N2-like polarization, enhanced NET formation, and higher levels of immunosuppressive mediators, such as PD-L1, TGF-β, and IL-6. In addition to established predictive biomarkers, such as PD-L1 expression and mismatch repair deficiency, emerging indicators, such as high tumor mutational burden, may further enhance immunotherapy stratification in TNBC [141]. Additionally, TNBC often features an immature or myxoid desmoplastic stroma. This stroma is highly dynamic and conducive to the recruitment of immune cells. Still, it can quickly become an immunosuppressive environment through interactions between TANs and CAFs, as well as through ECM remodeling and collagen cross-linking [96,115].

In contrast, there is limited direct evidence specifically addressing the abundance, phenotype, and function of TANs in triple-positive breast cancer (TPBC; ER+/PR+/HER2+). Most available data come from broader subtype analyses, which indicate higher TAN infiltration in HER2-positive and TNBC tumors than in hormone receptor-positive/HER2-negative cancers. For instance, higher HER2 was associated with neutrophils in HR-negative but not in HR-positive tumors, which may explain neutrophils’ well-known role in TNBC [142]. However, these analyses do not recognize TPBC as a distinct biological entity. As a result, the prognostic and functional significance of TANs in TPBC remains inadequately defined, highlighting a crucial gap in current knowledge and the necessity for subtype-specific investigations.

Interestingly, Soto-Perez-de-Celis et al. [41] conducted a retrospective study examining TANs in 105 stage I–III treatment-naive breast cancers and their relationship to tumor clinical aggressiveness. They found that 88% of TNBC and 53% of HER2-positive tumors were TAN-positive, compared to only 5% of hormone receptor–positive/HER2-negative tumors (*p* < 0.001). TAN positivity was strongly associated with hormone receptor negativity, HER2 expression, and higher histological grade. Multivariate analysis revealed hormone receptor negativity as the only independent predictor of TAN positivity (odds ratio 16.85, 95% CI 4.4–64.6, *p* < 0.0001). Based on the above they suggest that TANs are common in TNBC and may contribute to its clinical aggressiveness, underscoring the need for further investigation into their prognostic and therapeutic implications [41]. Meanwhile, a large, multi-cohort study by Wang et al. [42] demonstrated that the prognostic impact of tumor-infiltrating neutrophils (TINs) in treatment-naive breast cancer is highly compartment-specific. Across training, internal validation, and external validation cohorts, neutrophils localized within the tumor parenchyma, but not those in the stroma, emerged as a strong and independent predictor of poor survival, with hazard ratios exceeding 3.5 in all datasets. High parenchymal TIN density was significantly associated with adverse clinicopathological features, including higher histological grade, larger tumor size, lymph node and distant metastasis, advanced TNM stage, and enrichment in the TNBC subtype. Mechanistically, neutrophils promoted epithelial–mesenchymal transition (EMT) in breast cancer cells via TIMP-1, while EMT-activated tumor cells reciprocally enhanced neutrophil TIMP-1 secretion through CD90-dependent cell–cell contact, establishing a reinforcing pro-metastatic loop. In vivo, disruption of this axis through TIMP-1 neutralization or CD90 blockade significantly reduced metastasis. These findings identify parenchymal neutrophils as key drivers of EMT and metastasis, particularly in TNBC, and highlight TIMP-1/CD90 signaling as a potential therapeutic target [42].

Research has also shown that TNBC exhibits greater neutrophil infiltration, with N2 TANs demonstrating significant lipid accumulation. This specific metabolic characteristic makes N2-TANs, similar to TNBC tumor cells, particularly susceptible to ferroptosis, a regulated form of cell death that relies on iron and lipid peroxidation. A central finding of this research is the role of the protein FTH1 (ferritin heavy chain 1), which is overexpressed in N2-TANs and is targeted by the molecule CT-1. Targeting FTH1 activates feritinophagy, which, in turn, induces ferroptosis in both cancer cells and N2-TANs [143].

Lastly, data directly comparing triple-positive (TP) breast cancer (TPBC) and TNBC in untreated or pre-targeted therapy eras are limited. Available retrospective studies suggest that while both subtypes can exhibit aggressive clinicopathological features, differences in clinical outcomes are strongly influenced by treatment availability rather than intrinsic tumor malignancy. However, it is widely known that TNBC typically exhibits a more aggressive nature compared to all other breast cancer subtypes [144,145]. Negi et al. [146] were among the first to report that, although both TNBC and TPBC exhibit aggressive clinicopathological features, TNBC tumors display a more biologically aggressive behavior than TPBC tumors [147]. TNBC is associated with a younger age at diagnosis, more advanced stages of cancer, higher tumor grades, and increased rates of locoregional recurrence and metastasis compared to TPBC tumors. Notably, the comparatively better outcomes seen in TP breast cancer can be attributed mainly to receptor positivity, which allows for the use of endocrine and HER2-targeted therapies. Similarly, Kadi et al. [145] observed that the differences in prognosis between TNBC and TPBC are primarily influenced by treatment and clinical-pathological factors, rather than indicating that TNBC is intrinsically more malignant. The findings showed that while TNBC had higher mortality rates, it was not an independent predictor of overall survival after adjusting for other factors. The key determinants of patient outcomes were age at diagnosis, type of surgical treatment, and lymphovascular invasion. TNBC was also linked to a higher risk of local recurrence and showed more aggressive characteristics, with a nuclear pleomorphism score of 3 in 60.4% of cases, compared to 34.8% in TPBC (*p* < 0.001), and a high mitotic count, with a score of 3 in 48.9% of patients, compared to 14.2% [145]. In contrast, In a comparative study conducted by Alwan et al. [148], it was found that treatment-naive patients with HER2-positive breast cancer, TNBC, and TPBC exhibited notable differences. TNBC patients were significantly younger and tended to present with larger tumors. In contrast, HER2-enriched tumors were linked to more advanced disease stages, higher histological grades, and greater lymph node involvement. The authors concluded that the observed aggressiveness among these subtypes is due to differences in age at diagnosis and disease stage, rather than a uniform level of malignancy, underscoring the importance of molecular classification for prognostic purposes, especially in resource-limited settings.

In contrast to the immature, myxoid desmoplastic patterns frequently observed in TNBC, luminal breast cancers tend to exhibit more organized stromal architectures, which may partially restrain immune cell infiltration and contribute to distinct immunotherapy response profiles. Recent evidence indicates that, according to PAM50 classification, the luminal B subtype, rather than luminal A, exhibits features of activated adaptive immune responses. Tumors of the luminal B subtype display heightened immune microenvironment activation, suggesting that this group may derive greater benefit from immunotherapeutic approaches [149]. A recent study by Schmidt et al. [53] shows that TANs in early luminal breast cancer are a significant negative prognostic marker, challenging the traditional view that TANs primarily have antineoplastic (anti-cancer) effects in the initial stages of the disease. While the luminal subtype is generally considered less immunogenic, a higher density of CD66b+ neutrophils is directly associated with lower rates of Disease-Free Survival (DFS). The role of TANs in early luminal breast cancer extends beyond the tumor itself, impacting adjacent normal tissue and even lymph nodes that have not metastasized. Specifically, elevated levels of TANs in the stroma of lymph nodes are associated with a subgroup of patients who are at a higher risk of relapse or mortality. This finding indicates that cancer cells may attract neutrophils and influence their function beyond the confines of the traditional TME [53]. Lv et al. [150] also analyzed the TME of luminal B breast cancer, revealing its important prognostic and therapeutic implications. By integrating TCGA and GEO data and using CIBERSORT-based immune deconvolution, the authors developed a quantitative TME scoring system. High TME scores correlated with poorer patient outcomes and elevated expression of immune-related genes like *CXCL9*, *CXCL10*, *GZMB*, and *PDCD1LG2*. Additionally, tumors with high TME scores had a lower frequency of TP53 mutations, indicating distinct genomic-immune interactions. The TME score also predicted responsiveness to immune checkpoint inhibitors, showing its potential as a prognostic biomarker and a guide for immunotherapy in luminal B breast cancer. These findings underscore the urgent need for in-depth investigation of the TME as a determinant of immunotherapy efficacy, particularly in breast cancer, where variable immune checkpoint responses highlight the necessity for TME-informed biomarkers to better predict and optimize patient benefit from immune-based treatments [151,152].

To sum up, the prognostic significance of the stroma–tumor ratio (STR) varies across different molecular subtypes of breast cancer, highlighting distinct underlying biological mechanisms. In TNBC, pathways related to hypoxia, necrosis, and the immune response predominantly influence stromal effects. In contrast, fibrosis-associated processes are more relevant in luminal tumors. The status of stromal immune cells is a critical prognostic factor in TNBC. Additionally, assessing STR in combination with TILs represents a promising histopathological approach to improve patient stratification. By integrating STR and TILs into a unified scoring system, we can facilitate routine evaluation of TME features, ultimately serving as a robust, practical prognostic and predictive biomarker [153].

## 6. Therapeutic Prospects-Future Directions

**Figure 4:**
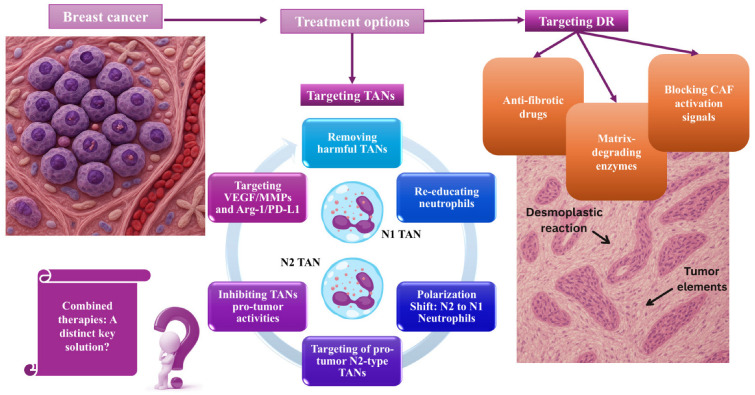
Therapeutic Targeting of TANs and DR in Breast Cancer: Strategies and Points of Intervention.

Our improved understanding of the essential roles of TANs and the DR in breast cancer, and of their interactive relationship as active partners, has identified new therapeutic targets [23,28]. Developing therapies that can target these elements, whether individually or, preferably, simultaneously, could be a significant objective to enhance patient prognosis and help prevent the development of resistance to existing treatments [23] (Figure 4).

### 6.1. Targeting TANs

Researchers are exploring strategies to mitigate the harmful effects of TANs [22,23].

#### 6.1.1. Removing Harmful TANs

Current strategies in breast cancer to block TAN recruitment or reduce intratumoral TAN populations have shown limited effectiveness thus far and remain primarily in clinical and preclinical studies [154,155,156]. A straightforward, practical approach could be to eliminate pro-tumorigenic neutrophils, possibly using antibodies that recognize specific markers on the TANs surface, as well as drugs that could affect their survival [21,27].

#### 6.1.2. Re-Educating Neutrophils

An alternative targeted strategy could focus on converting pro-tumorigenic N2 neutrophils back to their anti-tumor N1 form [21,22,23,25,27,34,96] use of drugs that block key signaling pathways, such as STAT3 or the administration of cytokines such as IFN-β that promote the beneficial N1 state, could be employed to achieve this goal f [21,22,23,25,27,34,96]. In studies using mouse models of breast cancer, researchers found that blocking TGF-β signaling not only reprogrammed TANs but also enhanced the efficacy of immune checkpoint inhibitors, such as anti-PD-1 and anti-CTLA-4 therapies [157,158]. These results indicate that combining TAN reprogramming with existing immunotherapies could strengthen anti-tumor responses [159].

#### 6.1.3. Targeting of Pro-Tumor N2-Type TANs

Neutrophils are the most abundant immune cells in the body, so their widespread removal or blockade could compromise overall immune function [82]. This makes the selective targeting of pro-tumor N2-type TANs a promising strategy for treating TNBC. N2-type TANs are particularly problematic because they significantly contribute to TNBC progression by promoting tumor growth, angiogenesis, immune suppression, and metastasis through mechanisms such as NET release. Therefore, it is crucial to develop innovative strategies and drugs that can selectively eliminate N2-type TANs without affecting the beneficial N1-type TANs. Liu et al. [143] introduced CT-1, a small molecule that effectively inhibits N2-type TANs at low doses (e.g., 4 μM in vitro and 5 mg/kg in vivo) while sparing N1-type TANs. These findings suggest that CT-1 could be a promising targeted agent to enhance cancer immunotherapy by specifically depleting pro-tumor N2-type TANs. Their study also highlights the high expression of FTH1 in both pro-tumor TANs and TNBC cells, indicating its potential as a therapeutic target for neutrophil-specific cancer therapies. CT-1, which targets FTH1, represents a novel therapeutic approach for TNBC by inducing ferroptosis in both TANs and TNBC cells. Targeting FTH1 to trigger ferroptosis in N2-type TANs and TNBC cells emerges as a compelling strategy for TNBC therapy, opening avenues for future clinical applications. Experimental data indicate that neutrophil ferroptosis contributes approximately 40% to the overall antitumor effect. This highlights that targeting both TANs and tumor cells is significantly more effective than focusing on the tumor alone. Consequently, the “dual ferroptosis” strategy via FTH1 offers a new perspective for managing TNBC by transforming the immunosuppressive microenvironment into one that promotes a therapeutic response [143].

#### 6.1.4. Inhibiting TANs Pro-Tumor Activities

This practice aims to weaken TANs by using drugs that inhibit their harmful functions [23,24,34]. Such therapies could include anti-VEGF agents and MMPs to block angiogenesis and tumor growth or Arg-1 or PD-L1 inhibitors to counteract their immunosuppressive effects [21,22,23,24,34,91]. In breast cancer, several clinical trials have investigated the role of angiogenesis inhibitors such as bevacizumab, ramucirumab, and tyrosine kinase inhibitors, with most yielding moderate results or fewer [160].

### 6.2. Targeting DR

Likewise, various strategies are being developed to dissolve or normalize the supporting fibrous layer [22] (Figure 4):

❖ Anti-fibrotic drugs: The aim of these agents may be to decrease the density and stiffness of the stroma. This could be achieved by targeting the cells that contribute to its formation [26,27,101]. For example, inhibitors of the TGF-β pathway could prevent the activation of fibroblasts, potentially leading to enhanced drug delivery and improved access for immune cells [22,23,24,26,27,101,121].
❖ Matrix-degrading enzymes or nanoparticles: This design could utilize enzymes such as collagenases to physically break down components of the ECM therefore, the goal is to eliminate the natural barrier created by DR, allowing passage for drugs and immune cells [22,23,26,101]. Recent studies also suggest the use of matrix-degrading soft-nanoplatform as an alternative treatment method in breast cancer patients. Lu et al. [161], investigated the role of matrix-degrading soft nanocapsules (HSA/HAase SNCs), which enzymatically loosen the ECM barriers in tumors, thereby enhancing the performance of nanotherapy. These HSA-hyaluronidase capsules were found to be biocompatible, exhibiting no cytotoxicity or hemolysis. They demonstrated a tumor-cell uptake that was approximately 1.4 times higher than that of stiffer counterparts and showed enhanced penetration in 4T1, CT26, and Pan02 spheroids. When loaded with chlorin e6 (HSA/HAase@Ce6), this platform produced increased ROS, achieved deeper distribution within tumors, and significantly suppressed tumor growth in breast cancer mouse models. RNA sequencing of the treated tumors revealed an enrichment of ECM-degradation pathways, confirming the underlying mechanism. Overall, ECM-degrading soft nanocapsules represent a promising strategy to overcome stromal barriers and enhance the efficacy of nanomedicines, such as photodynamic therapy.
❖ Blocking CAF activation signals: Targeting CAFs through specific surface molecules, primarily fibroblast activation protein (FAP) [26,27], presents a promising approach to reducing the DR and remodeling the tumor stroma [22,23,26,27,100,121]. Inhibiting FAP activity may decrease overall stromal fibrosis and stiffness, thereby enhance drug delivery and improve antitumor immunity. Various therapeutic strategies are currently under development, including FAP inhibitors, small molecules, and antibodies targeting FAP, as well as FAPα-CAR-T cells, which are progressing through preclinical studies. Notably, the humanized anti-FAP antibody sibrotuzumab has shown acceptable safety but limited efficacy in early cancer trials. In a recent study conducted by Honda et al. [162] the researchers investigated the relationship between different populations of CAFs and the exclusion of immune cells in breast tumors. They found that interactions among various CAF subsets, such as EMILIN1 from IFNγ-induced CAFs, influence TGF-β activity in the TME. This research suggests the need for therapies that target biological processes rather than focusing on specific CAF subtypes [162]. Additionally, combining FAP-directed therapies with chemotherapy, radiotherapy, or immunotherapy may enhance treatment responses while minimizing off-target toxicity by leveraging the stroma’s selectivity. Overall, FAP serves as a viable therapeutic target with significant potential for translation into clinical practice [163].

### 6.3. Combined Therapies: A Clinically-Grounded Perspective

By examining the deep-seated connection between TANs and the desmoplastic stroma of tumors, it becomes evident that a multifaceted combination therapy targeting the tumor, stroma, and immune system simultaneously would be significantly more effective [27]. From a clinical standpoint, a promising and practical approach could involve combining drugs that degrade the fibrotic stroma with those that activate the immune system [22]. For instance, using an antifibrotic agent, such as a TGF-β inhibitor, could physically disrupt the stromal barrier [26]. This disruption may enable the more effective delivery of traditional chemotherapy and facilitate better infiltration of cytotoxic T-cells into the tumor, thereby enhancing the efficacy of immune checkpoint inhibitors, such as anti-PD-L1 agents [27]. However, this strategy poses a significant challenge, as N2-polarized TANs are known to express PD-L1, which directly links them to the immunosuppressive environment these immunotherapies aim to counteract [21,22,23]. Another promising therapeutic target is the STAT3 signaling pathway, which serves as a master regulator in the activation of both CAFs and pro-tumor N2 TANs [23]. Additionally, extensive research has focused on therapies that target FAP to neutralize CAFs, as well as the use of angiotensin inhibitors to decompress the tumor’s blood vessels. Both strategies aim to overcome the resistance posed by stromal tissue while enhancing the effectiveness of chemotherapy and immunotherapy [22,26].

Despite the promise of such combination strategies, their efficacy remains constrained by the physical, cellular, and metabolic barriers of the TME, underscoring the need for next-generation approaches capable of precisely reprogramming the tumor immune microenvironment (TIME) [164]. The TIME adopts an immunosuppressive structure by recruiting regulatory immune cells and activating inhibitory cytokines and immune checkpoint pathways. This process limits effective antitumor immunity [165,166,167,168]. Significantly, therapeutic interventions can also reshape the TIME. For example, targeted therapies can induce adaptive immunosuppressive states, which may ultimately lead to treatment failure [169]. Concurrently, metabolic reprogramming within the TME, characterized by enhanced glycolysis, hypoxia, and lactic acid accumulation, impairs cytotoxic immune cell function while promoting epithelial–mesenchymal transition and metastasis. Metabolic reprogramming in the TME is characterized by increased glycolysis, low oxygen levels (hypoxia), and lactic acid accumulation [170]. These changes impair the function of cytotoxic immune cells while promoting epithelial–mesenchymal transition and metastasis. It is therefore clear that these characteristics highlight the limitations of single-target therapeutic strategies and underscore the need for integrative approaches that account for the spatial, cellular, and metabolic complexity of the TME. In this context, advanced analytical methods like single-cell sequencing and mass cytometry (CyTOF) are powerful tools for analyzing therapy-resistant cellular networks and immune-stromal interactions, highlighting the TME as a vital therapeutic target [171].

Insights from systemic analyses have spurred the development of next-generation therapeutic platforms to overcome both physical and immunological barriers within the TME. Nanomedicine and exosome-mediated delivery systems have shown particular promise in modulating the diverse TME and enhancing antitumor immunity [172,173]. For example, matrix-degrading soft nanoplatforms enzymatically loosen the stiff ECM that limits drug penetration, thereby improving intratumoral delivery and increasing the efficacy of photodynamic therapies. At the same time, emerging strategies that induce immunogenic cell death (ICD), such as small molecules targeting ferritin heavy chain 1 (FTH1) to trigger ferritinophagy-mediated ferroptosis, offer dual therapeutic benefits [174]. These strategies not only eliminate immunosuppressive components but also activate systemic antitumor immune responses in both cancer cells and pro-tumor N2 tumor-associated neutrophils. Nanoparticle-based delivery systems further enhance these effects by enabling targeted tumor accumulation, deep tissue penetration, improved cellular uptake, and controlled release of ICD inducers, while also allowing the co-delivery of immunomodulatory agents to counteract the immunosuppressive tumor environment [172]. Although these approaches have shown strong efficacy in preclinical models, their clinical translation faces challenges related to tumor-specific drug distribution, the simplicity of nanoparticle design, quality control, and a limited understanding of the mechanisms behind ICD-driven immune activation.

Integrating ICD-based therapies with exosome-mediated strategies offers a promising way to limit metastasis and overcome treatment resistance in breast cancer subtypes like TNBC. Exosomes serve as effective delivery vehicles, modulating the tumor microenvironment, inhibiting angiogenesis, and suppressing metastatic niches. Stimuli-responsive nanoparticles further enhance precision by navigating the dense stroma and high interstitial pressure typical of breast tumors [175]. Notably, exosome membrane–coated biomimetic nanoparticles have demonstrated preferential lung accumulation, efficient gene silencing, and significant suppression of postoperative TNBC lung metastasis in vivo [173]. These platforms not only have direct anti-metastatic effects but may also transform the immunosuppressive TME into an active state, enhancing the effectiveness of immune checkpoint therapies. Additionally, integrating these delivery systems with tools such as single-cell sequencing provides a robust framework for patient stratification and personalized treatment, aiming to overcome therapy resistance and prevent cancer progression.

### 6.4. Clinical Implications and Translational Perspectives

While the molecular mechanisms discussed above provide a strong biological basis for targeting tumor-associated neutrophils and desmoplastic stroma, it is crucial to recognize that not all proposed strategies have translated into clinical benefits. For instance, despite compelling preclinical evidence supporting the inhibition of MMPs to limit ECM remodeling and invasion, broad-spectrum MMP inhibitors have failed in multiple clinical trials. This failure has been attributed to limited efficacy, poor patient selection, and dose-limiting toxicities; therefore, these inhibitors are not included in current cancer treatment regimens [176]. Recent studies have investigated the use of nanotechnology to enhance the targeting of MMPs [177]. This focus arises from the failure of broad-spectrum MMP inhibitors, such as marimastat, in clinical trials due to their limited effectiveness and off-target toxicities caused by nonspecific zinc chelation [178]. Preclinical nanoplatforms include amyloid-derived peptide nanofibers linked to doxorubicin that also inhibit MMP activity [179], metallofullerenol nanoparticles that allosterically block MMP-2/9 synthesis [180], thermosensitive liposomes delivering marimastat [181], and oligonucleotide-loaded peptide or polymeric carriers targeting MMP expression [182,183]. While these approaches have shown promise in reducing tumor growth and invasiveness in experimental models, they remain at the preclinical stage and have yet to demonstrate clinical benefits [177].

In contrast, chemotherapy has proven to be the most successful treatment meth-od in breast cancer. Importantly, much of the current evidence describing the stromal composition and immune infiltrates in TNBC is derived from tumors exposed to cytotoxic chemotherapy regimens, such as taxanes and platinum-based agents, which induce extensive cell death, necrosis, and tissue injury. These treatment-induced effects can trigger a robust secondary inflammatory response characterized by stromal remodeling and pronounced neutrophil recruitment. Consequently, the elevated TAN levels observed in TNBC may partially reflect chemotherapy-driven inflammation rather than intrinsic tumor biology. Supporting this interpretation, increased neutrophil infiltration following chemotherapy has been associated with adverse therapeutic responses and poorer outcomes in breast cancer [184,185]. To accurately comprehend the cause-and-effect relationship, it is important to consider evidence from chemotherapy-naive patient groups. Recent studies by Kakumoto et al. [39] and Schmidt et al. [53] have shown that TAN density remains a significant and independent prognostic factor, even in the absence of prior systemic treatment. These findings highlight the biological role of TANs in disease progression and emphasize the need to consider treatment exposure when interpreting differences in the microenvironment across various breast cancer subtypes.

Recent single-cell studies indicate that chemotherapy itself substantially reshapes the immune and stromal landscape of TNBC. Patyseva et al. [186] demonstrated that non-responders to neoadjuvant chemotherapy exhibit early immune dysregulation and expansion of immunosuppressive myeloid populations, which were validated as predictors of poor treatment response. They concluded that elevated neutrophil and myeloid signatures in treated TNBC may, in part, reflect therapy-induced inflammatory remodeling rather than intrinsic tumor clinical aggressiveness.

Moreover, immune checkpoint inhibitors that target PD-1/PD-L1 have proven to be an effective treatment option in breast cancer, especially for specific subsets of TNBC. However, anti-PD-1 therapies have been studied more extensively than anti-PD-L1 therapies in breast cancer [187]. Agents like pembrolizumab and atezolizumab have shown survival benefits in biomarker-defined populations. Regarding pembrolizumab, the KEYNOTE-012 study showed early clinical results (overall response rate, ORR, 18.5%) in PD-L1–positive metastatic TNBC [188], but pembrolizumab combined with chemotherapy significantly improved outcomes in PD-L1–positive metastatic TNBC, leading to FDA approval in November 2020 [189]. thus, pembrolizumab is clinically beneficial when used in combination with chemotherapy and in biomarker-selected (PD-L1–positive) TNBC patients, not as monotherapy. Today, pembrolizumab is an immune checkpoint inhibitor used in both early-stage neoadjuvant and metastatic settings, primarily for TNBC [190]. Additionally, results from the IMpassion130 [191] and KEYNOTE-355 [192,193] trials showed that when atezolizumab or pembrolizumab is combined with chemotherapy, there is a significant progression-free survival benefit in advanced TNBC with positive PD-L1 expression, but not in tumors with negative PD-L1 expression. Likewise, antibody-drug conjugates such as sacituzumab govitecan (commonly known as Trodelvy, used in subtypes such as TNBC, hormone receptor–positive, and HER2-negative breast cancer) and trastuzumab deruxtecan (widely known as T-DXd, used in HER2-positive or HER2-low breast cancer) are also used in breast cancer therapy [190]. Recently, in the phase III OptiTROP-Breast01 trial, Sacituzumab tirumotecan significantly improved progression-free survival compared with chemotherapy in heavily pretreated metastatic TNBC (median PFS 6.7 vs. 2.5 months; HR 0.32, *p* < 0.00001), with concurrent overall survival benefit and a manageable safety profile [194]. Moreover, PARP inhibitors have been validated in BRCA-mutated breast cancer, improving progression-free survival in multiple clinical trials. For instance, in the phase III OlympiA trial, adjuvant olaparib significantly improved invasive and distant disease–free survival in patients with high-risk, HER2-negative early breast cancer carrying germline BRCA1/2 mutations (3-year IDFS 85.9% vs. 77.1%; HR 0.58; *p* < 0.001), with a favorable safety profile [195], while in a phase I dose-expansion study, talazoparib monotherapy demonstrated clinically meaningful activity in Japanese patients with germline BRCA1/2–mutated, HER2-negative advanced breast cancer, achieving an objective response rate of 57.9% and a median progression-free survival of 7.2 months, with manageable hematologic toxicity [196]. These examples underscore that, while mechanistically driven therapies such as MMP inhibitors have failed clinically, other novel agents have achieved robust evidence of benefit, highlighting the evolving therapeutic landscape in breast cancer.

## 7. Conclusions

The ΤΜΕ plays a crucial role in the progression of breast cancer, with TANs and the DR serving as interdependent drivers [21,22,23,26,27,28]. The interaction between these elments influences invasion, metastasis, and response to therapy. The common occurrence of immature DR patterns, such as myxoid stroma, in high histological grade tumors and elevated TAN infiltration suggests a functional stromal–immune axis that may contribute to tumor progression and adverse clinicopathological behavior [21,22,25].

Moving forward, key areas of focus should include:

(a) Mechanism-based characterization of TAN plasticity across different DR states. This involves using spatial and longitudinal approaches to define the cytokine (e.g., TGF-β/IL-6) and biomechanical factors (such as stiffness and ECM composition) that are associated with the induction of pro-tumor neutrophil programs.
(b) The development and validation of composite biomarkers that integrate the maturity of the DR (ranging from myxoid to keloid-like to mature), hyaluronan/collagen metrics, and TAN phenotype/NETosis measurements. Such biomarkers could facilitate patient stratification and support real-time assessments of pharmacodynamic responses [22,23,26,28,37,96,107].
(c) The rational design of clinical trials to evaluate strategies that modulate TAN function (such as reprogramming from N2 to N1 phenotypes and constraining NET formation) in combination with interventions that normalize the stroma (e.g., anti-fibrotic or ECM-modulating treatments) and immunotherapy. These approaches may help disrupt the TAN–DR feedback loop and potentially enhance the durability of therapeutic responses [21,22,23,24,28,35,37]. The integration of these context-specific strategies may support the translation of TAN–DR biology into actionable biomarkers and inform the development of more effective therapeutic approaches for clinically aggressive breast cancer [23,28].

Furthermore, advancing neutrophil-based immunotherapy for breast cancer will likely require a carefully structured and mechanism-driven approach. Such efforts should aim to:

➢ Tailor therapeutic interventions based on tumor subtype and individual immune context using biomarkers of TAN density, polarization status, cytokine milieu, and NET activity.
➢ Utilize neutrophil plasticity to enhance N1-like phenotypes by selectively modulating pathways such as CXCR2–CXCL8, TGF-β/STAT3, and IFN-β.
➢ Explore combinatorial strategies integrating TAN-directed approaches with complementary immunomodulators (e.g., PD-1/PD-L1 blockade), anti-angiogenic agents, and cytotoxic therapies to remodel the TME and enhance antitumor immune responses.
➢ Develop and carefully evaluate NET-targeted interventions that aim to reduce metastatic dissemination while preserving essential antimicrobial functions.
➢ Further elucidate the systems-level interactions between neutrophils and tumor, stromal, endothelial, and myeloid/lymphoid compartments.
➢ Employ advanced human-relevant models such as humanized mouse systems, organoids, and high-resolution imaging to analyze TAN heterogeneity and dynamics in situ.
➢ Incorporate standardized pharmacodynamic monitoring of neutrophil infiltration, activation state, polarization, and NETosis into clinical trial designs to guide dosing, scheduling, and combination strategies.
➢ Emphasize safety considerations, ensuring selective modulation of intratumoral neutrophil functions while minimizing the risks of infection, systemic inflammation, and immune-related toxicities [159].

### Limitations and Future Research Directions

Despite increasing interest in the role of tumor-associated neutrophils and desmoplastic stroma in breast cancer, several significant limitations remain. A substantial proportion of mechanistic insights derives from murine models, which do not fully recapitulate the complexity, heterogeneity, and treatment history of human breast tumors. Additionally, the lack of standardized methods for identifying TANs, ranging from morphology-based assessments to immunohistochemical and single-cell transcriptomic approaches, creates challenges for cross-study comparisons and may lead to conflicting clinical associations. Moreover, TAN phenotypes are highly dynamic and influenced by treatment exposure, stromal composition, and spatial localization. This underscores the necessity for longitudinal and spatially resolved analyses in both treatment-naive and treated patient cohorts. Future research should focus on establishing standardized TAN classification frameworks, integrating spatial multi-omics, and validating findings in well-annotated human clinical samples. Emphasis should be placed on distinguishing therapy-induced inflammatory changes from intrinsic tumor biology. Further, understanding context-specific TAN-stroma interactions could inform biomarker development and guide rational therapeutic targeting.

## Figures and Tables

**Table 1:** Key Pro-Tumorigenic Mechanisms of N2 TANs and the DR.

Component	Key Pro-Tumorigenic Mechanisms/Effects	Details	References
N2 TANs	Immunosuppression	Immunosuppressive mediators such as Arg-1, ROS, and PD-L1.	[22,23,34,91,133]
	Angiogenesis	Pro-angiogenic factors including VEGF, MMP-9 (which releases ECM-bound growth factors), and the peptide Bv8.	[21,22,23,24,25,28,34,35,53]
	Metastasis	Remodeling the ECM with proteases, forming pre-metastatic niches, inducing EMT in cancer cells, and promoting tumor cell survival.	[21,22,23,24,25,28,34,35,53]
	Therapeutic Resistance	Chemotherapy resistance, protection of cancer cells from apoptosis. Sequestration of drugs and activation of survival pathways.	[22,24,28,34,35]
DR	Physical Barrier	Prevention of chemotherapy drugs and of immune cells like T-cells and neutrophils.	[22,25,26,27,28,29,96,100,101,121]
	Increased Tumor Stiffness & Invasion	ECM deposition promotes tissue stiffness. Tumor cell proliferation, migration, and invasion, usually through mechanosensors like integrins.	[22,25,29,97,98,100,101]
	Pro-tumorigenic Signaling of CAFs	Growth factors (e.g., PDGF, TGF-β1, FGF) and activation of myofibroblasts and help tumors grow.	[21,22,28,29,99]
	ECM Remodeling	Degradation of the ECM. Physical pathways for tumor cells to infiltrate the tissues and also access blood vessels.	[21,22,25,27,28,29,98,100,101]
	Enhancement of Therapeutic Resistance	Barrier to drug delivery. CAFs can secrete factors that protect cancer cells from therapies.	[2,5,6,8,9,16,20,24]

Note: Arg-1: Arginase-1; ROS: Reactive Oxygen Species; PD-L1: Programmed Death-Ligand 1; VEGF: Vascular Endothelial Growth Factor; MMP-9: Matrix Metalloproteinase-9; ECM: Extracellular Matrix; EMT: Epithelial-Mesenchymal Transition; PDGF: Platelet-Derived Growth Factor; TGF-β1: Transforming Growth Factor beta-1; FGF: Fibroblast Growth Factor; CAFs: Cancer-Associated Fibroblasts; MMPs: Matrix Metalloproteinases.

## Data Availability

The data presented in this study are available in the tables and figures above.
